# Public Knowledge, Awareness, Attitudes, and Practices Regarding Seizure Attacks Among People of Makkah City

**DOI:** 10.7759/cureus.32485

**Published:** 2022-12-13

**Authors:** Abdullah A Tawakul, Atheer A Alqurashi, Shahad A Altayyar, Ashwaq H Bugis, Fadi S Althobaiti, Khalid M Almatrafi, Rami M Algahtani, Ahmad A Imam, Omar M Babteen

**Affiliations:** 1 Department of Internal Medicine/Neurology, College of Medicine, Umm Al-Qura University, Makkah, SAU; 2 Department of Medicine and Surgery, College of Medicine, Umm Al-Qura University, Makkah, SAU; 3 Department of Internal Medicine, College of Medicine, Umm Al-Qura University, Makkah, SAU; 4 Department of Physiology, College of Medicine, Umm Al-Qura University, Makkah, SAU

**Keywords:** attitudes, awareness, knowledge, makkah, seizure, epilepsy

## Abstract

Objectives: Seizures can occur as a result of a variety of health issues. Epilepsy is a common neurological disease and it is the most prevalent cause of seizures. Epileptic patients might experience a seizure attack at any moment. The aim of this study is to assess public knowledge, awareness, attitudes, and practices toward seizure attacks among residents of Makkah city.

Methods: A cross-sectional study was conducted utilizing an online questionnaire, which was distributed through various social media platforms. The questionnaire consisted of five parts, taking sociodemographic characteristics into consideration, and evaluating knowledge, awareness, attitudes, and practices among the general population of Makkah city.

Results: A total of 401 participants completed the study questionnaire: 280 (69.8%) participants were females and 121 (30.2%) were males. Overall knowledge regarding epilepsy among the study participants was evaluated. A total of 132 (32.9%) participants had a good level of knowledge, while 269 (67.1%) exhibited poor knowledge. In addition, students had significantly better knowledge (44.7%) than individuals who were employed, retired, or unemployed (27.7%), (P=.004). Furthermore, participants who had previously heard about epilepsy were more knowledgeable (34.3%) than those who had not (P =.041). Additionally, participants who attended a course on seizure control (46.7%) had significantly better knowledge than those who did not (31.2%), (P=.037).

Conclusion: This study revealed that most of our sample of Makkah city residents had poor overall knowledge of epilepsy and seizure attacks. A health education program and awareness campaigns could help improve this lack of knowledge in Makkah city.

## Introduction

A seizure is an occurrence of signs and/or symptoms due to abnormal, hyperexcitable activity, or synchronous neuronal activity in the brain. It can cause alterations in behavior, movements, feelings, and consciousness. Various types of seizures could occur in different parts of the brain and could be localized, involving just one body part, or generalized, involving the entire body [1]. Seizures can stem from several health conditions. The most common reason for seizures is epilepsy. However, not everyone who experiences seizures has epilepsy. Seizures can be provoked by several impediments such as alcohol withdrawal, brain injury through childbirth, brain infection like meningitis drug abuse and withdrawal, and electrolyte disturbance. Management of seizures does not imply restricting movement forcibly, but rather limiting damage or complications during the seizure. Healthcare professionals should be equipped to identify, diagnose, and manage non-epileptic seizures and epilepsy, and should ensure that patients receive adequate information about their disorders and how to manage it [2,3].

According to the Epilepsy Foundation of America, 2017, “Epilepsy is not rare, it’s more common than autism, Parkinson’s disease, multiple sclerosis, and cerebral palsy combined. One in 26 people will develop epilepsy at a certain point in their life [4]”. Moreover, the World Health Organization’s 2010 Global Burden of Disease study positions epilepsy as the second most burdensome neurological problem worldwide. As reported by a study in 2017, the lifetime prevalence of epilepsy was 7.6 per 1000 persons, and the incidence rate was 67.77 per 100,000 persons [5]. The literature review showed varying levels of public awareness, knowledge, and attitude towards epilepsy and seizure among various societies. A series of research studies were conducted in the Kingdom of Saudi Arabia, investigating public awareness and attitudes concerning seizures and epilepsy. One of these studies assessed the knowledge of seizure first aid among schoolteachers in the Makkah region. The majority of these teachers (55%) lacked adequate seizure first aid training and knowledge. However, the study was limited to female teachers, and studies were not conducted to evaluate the levels of public awareness, knowledge, and attitude toward epilepsy and seizures in the general population of Makkah city, considering its high cultural diversity due to Omrah and Hajj, which is considered one of the world’s largest human gatherings [6]. Another study was conducted in Jeddah and Al-Kharj, measuring public perception and attitude toward epilepsy. The results suggest that awareness about epilepsy in the public should be raised to avoid a negative impact on patients. There are several misconceptions identified, predominately in the management and etiology of the disease [7,8]. Additional studies done in Al-Taif and Tabuk measured the schoolteacher attitude, which showed inadequate practices when looking after an epilepsy patient during a seizure attack [9,10]. It is apparent that evaluating public awareness and behavior among the public, schoolteachers, and healthcare practitioners is the first step to promoting the Ministry of Public Health's ongoing efforts to establish a local or national epilepsy management program. Several international studies have shown a lack of understanding among the public, schoolteachers, and healthcare practitioners concerning seizures and the need for increasing awareness. Interestingly, recent studies showed an increased level of awareness of epilepsy in Western countries such as the United States and Italy, while more negative attitudes were seen in Asian countries, such as India and Malaysia [10]. Inappropriate seizure interventions have been reported in these countries such as spraying water over the patient's face, holding their tongue, or fixing their position. In addition to providing no direct benefit to the patient, these acts may cause significant harm to the seizing patient, or the person attempting to assist them [11].

## Materials and methods

Study design

The study design consists of a questionnaire for a descriptive cross-sectional study conducted between March and April 2022. The study aims to assess public knowledge, awareness, attitudes, and practices toward seizure attacks, among people living in Makkah city.

Study population

The study was conducted via an online questionnaire and our target sample was public adults aged 18 years and older and Arabic-speaking individuals who live in Makkah city. This study excluded individuals less than 18 years of age and non-Arabic speaking individuals.

Sampling methodology

The study was conducted on the sample public from the 1st of March to the 1st of April 2022 in Makkah city, Saudi Arabia. Consent was obtained from the participants before they complete the questionnaire. According to the Raosoft calculator (Raosoft Inc., Seattle, WA), the sample size should not be less than 377 participants. The questionnaire form was approved by two certified neurologists and was shared on social media platforms. The form consists of five parts. The first part includes sociodemographic characteristics: age, sex, marital status, educational level, occupation, and monthly income. The subsequent part is made of five yes/no inquiries evaluating epilepsy awareness. The third part evaluates the knowledge of epilepsy that formed of four multiple choice questions, and three answers in form of one close-ended question, either: ‘yes’, ‘no’, and ‘I do not know’. The fourth part assesses the participants’ attitudes toward epilepsy and is composed of 10 questions with the following answer choices: ‘agree’, ‘disagree’, and ‘neutral’ [8]. The last section includes eight different possible actions toward epilepsy during the seizure attack. There are only two correct actions to be taken when witnessing a convulsion: ‘putting the patient on their side’ and ‘calling the ambulance’ [12]. Data were collected from any participant who meets our criteria. Electronic data collection forms did not show any nominative information. Data was entered automatically into an excel sheet. After verification, data were transferred to SPSS software, version 22.0 (IBM Corp., Armonk, NY) for analysis. A pilot study was done on eight randomly selected middle-aged participants to check the reliability and clarity of the questions.

Data analysis

After the extraction of the data was done, we revised and also coded, and fed it to the statistical software IBM SPSS version 22.0. A P-value less than 0.05 was considered statistically significant and all statistical analyses were done using two-tailed tests. For knowledge items and awareness items, scored one point for each correct answer, and we calculated for each knowledge item the total summation of the discrete scores of the different items. A participant with a score of less than 60% of the total score was considered to have poor knowledge, while a score of 60% or more of the total was considered good knowledge. Based on frequency and percent distribution, the descriptive analyses were done for all variables, including the participants’ age, gender, education level, family history of epilepsy, history of attending training courses for epilepsy, and observing a patient during an epilepsy fit. Participants’ knowledge items including general knowledge, causes, and treatment of epilepsy were also tabulated and graphed. Participants' practices and attitudes toward epilepsy were also tabulated and graphed. We used cross-tabulation to assess factors associated with the public knowledge level with regard to epilepsy and its treatment. All the relations were tested using the exact probability test and also Pearson’s Chi-squared test for the small frequency distributions.

Ethical part and confidentiality

Consent was obtained from each participant through the questionnaire. Ethical approval was sought from the Biomedical Ethics Committee of Umm Al-Qura University (UQU) (NO: HAPO-02-K-012-2022-03-1015). Study activities were not started until approval was obtained.

## Results

A total of 401 participants completed the online study questionnaire. Participants' ages ranged from 18 to 59 years, with a mean age of 30.1 ± 12.8 years old. A total of 280 (69.8%) participants were females. In terms of marital status, 195 (48.6%) participants were single, while 189 (47.1%) were married. As for educational level, 303 (75.6%) were university graduates, 177 (44.1%) were working, 101 (25.2%) were unemployed, and 123 (30.7%) were students. A monthly income of less than 5000 Saudi Rial (SR) (1329 USD) was reported for 78 (19.5%) participants, 113 (28.2%) had a monthly income of 5000-10000 SR (1329-2659 USD), while 57 (14.2%) had a monthly income of more than 20000 SR (5319 USD). Family history of epilepsy was reported among 70 (17.5%) participants, 45 (11.2%) attended a course on seizure control, and 225 (56.1%) observed a patient suffering from an epileptic fit (Table 1).

**Table 1:** Personal characteristics of study participants, Makkah city, Saudi Arabia SR: Saudi Rial

Personal data	No	%
Age in years		
18-30	200	49.9%
31-49	162	40.4%
50+	39	9.7%
Gender		
Male	121	30.2%
Female	280	69.8%
Marital status		
Single	195	48.6%
Married	189	47.1%
Divorced / widow	17	4.2%
Educational level		
Below secondary	22	5.5%
Secondary	76	19.0%
University / above	303	75.6%
Employment		
Not working / retired	101	25.2%
Student	123	30.7%
Working	177	44.1%
Monthly income		
< 5000 SR	78	19.5%
5000-10000 SR	113	28.2%
10001-15000 SR	89	22.2%
15001-20000 SR	64	16.0%
> 20000 SR	57	14.2%
Family history of epilepsy		
Yes	70	17.5%
No	331	82.5%
Have you ever attended a course on seizure control?		
Yes	45	11.2%
No	356	88.8%
Have you ever seen a patient suffering from an epileptic fit?		
Yes	225	56.1%
No	176	43.9%

Out of the total, 68.3% of the participants know that epilepsy can result from neurological diseases, and 34.2% believe it may be a hereditary disease. Moreover, 15.2% of participants thought epilepsy might be due to medications, and only 7% thought it might be due to toxins. As for their knowledge of the clinical symptoms of epilepsy, 93.5% chose convulsions, 65.1% chose brief loss of consciousness, 53.1% chose mouth foaming, and 23.2% chose changes in behavior. Regarding the right action during an epileptic fit, 82.1% decided it is necessary to call the ambulance, and 55.1% were aware that they should tilt the patient on their side. As for the treatment of epilepsy, 73.6% of study participants were aware of medical treatments, 14.2% chose surgical treatment, while only 4% chose herbs. Additionally, 19.7% of the participants were aware that surgery is an option to treat epilepsy in intractable cases (Table 2).

**Table 2:** Knowledge regarding epilepsy among study participants, Makkah city, Saudi Arabia

Knowledge items		No	%
Causes of epilepsy	Neurological diseases	274	68.3%
	Genetic Disease	137	34.2%
	Medicines and toxins	61	15.2%
	Psychological diseases	112	27.9%
	Brain disease	218	54.4%
	Psychological or emotional stress	109	27.2%
	Infection	28	7.0%
	Evil spirits	51	12.7%
	Envy	86	21.4%
	Haematological diseases	11	2.7%
	Do not know	40	10.0%
Clinical symptoms of epilepsy	Convulsions	375	93.5%
	Brief loss of consciousness	261	65.1%
	Mouth foam	213	53.1%
	Changes in behaviour	93	23.2%
	Screaming	76	19.0%
	Do not know	15	3.7%
Right action during epileptic fit?	Tilt the patient to the side position	218	55.1%
	Attempting to pull the patient's tongue out to prevent him from swallowing his tongue	108	27.3%
	Call an ambulance	325	82.1%
	Hold the patient tightly and try to stop the shivering	93	23.5%
	Placing a hard object in the patient's mouth (other than a cloth)	52	13.1%
	Splashing water on the patient's face	47	11.9%
	Others	3	.8%
	Don't do anything and just watch	17	4.3%
Treatments of epilepsy	Drugs	295	73.6%
	Ruqia & Quraan	151	37.7%
	Surgical treatment	57	14.2%
	Herbs	16	4.0%
	Ironing	10	2.5%
	Epilepsy untreatable	31	7.7%
	Do not know	79	19.7%
Did you ever know that surgery is an option to treat epilepsy for uncontrollable cases?	Yes	79	19.7%
	No	226	56.4%
	Not sure	96	23.9%

A total of 81.5% agreed that women with epilepsy could marry and 79.1% agreed that children with epilepsy could succeed in public schools and do not need their own schools. A total of 77.1% reported that people with epilepsy should have equal job opportunities compared to people without epilepsy. A total of 76.1% of participants think that women with epilepsy can have a normal pregnancy. Finally, 37.2% would not mind if one of their children or a member of their family married a person with epilepsy, and only 1.5% think epilepsy is infectious (Table 3). 

**Table 3:** Attitude towards epilepsy and its treatment among study participants, Makkah city, Saudi Arabia

Attitude	Agree		Disagree		Not sure	
	No	%	No	%	No	%
Epilepsy is infectious	6	1.5%	366	91.3%	29	7.2%
Epilepsy is a type of madness	54	13.5%	285	71.1%	62	15.5%
Women with epilepsy can marry?	327	81.5%	15	3.7%	59	14.7%
Women with epilepsy can give birth?	305	76.1%	15	3.7%	81	20.2%
Children with epilepsy can succeed in public schools and do not need their own schools	317	79.1%	23	5.7%	61	15.2%
People with epilepsy should have equal job opportunities with people without epilepsy	309	77.1%	24	6.0%	68	17.0%
I wouldn't mind if one of my children or a member of my family married a person with epilepsy	149	37.2%	87	21.7%	165	41.1%

**Figure 1:**
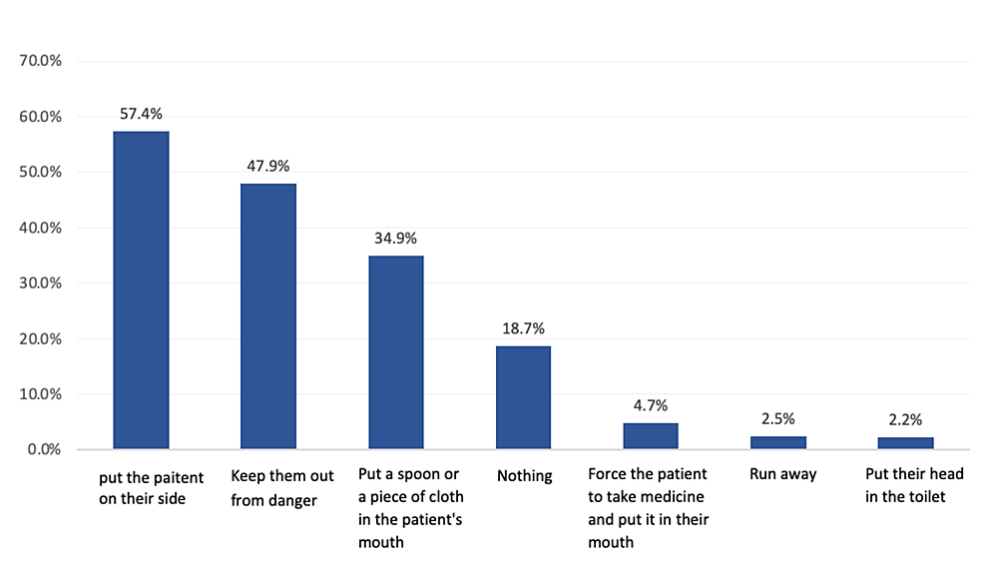
The actions that will be taken while confronting a person having an epileptic fit as reported by study participants

Regarding the actions during the attack, most participants (57.4%) reported that tilting the patient on their side (which is the correct action) is important; 47.9% chose ‘keeping patients away from danger’, 34.9% chose ‘placing a spoon or cotton piece in the patient's mouth’, while 18.7% reported they would do nothing and 2.5% would run away (Figure 1).

**Figure 2:**
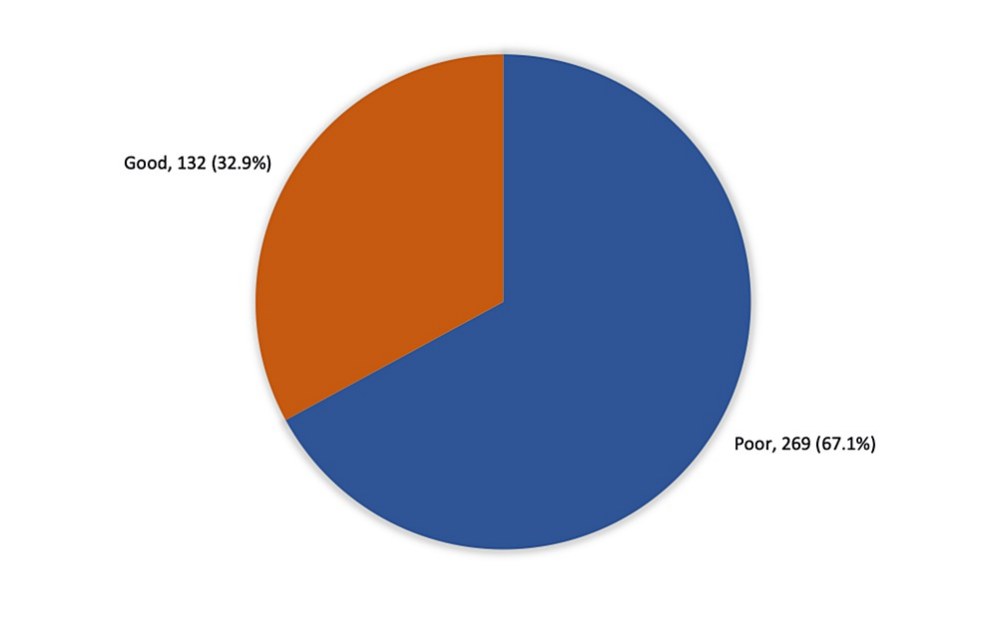
Overall participants' knowledge regarding epilepsy and its treatment among study participants in Makkah city, Saudi Arabia

A total of 132 (32.9%) participants had a good knowledge level of epilepsy, while 269 (67.1%) had poor knowledge (Figure 2).

Students had significantly better knowledge (44.7%) than individuals who were employed, retired, or unemployed (P=.004). Furthermore, participants who had heard about epilepsy previously were significantly more knowledgeable (34.3%) than those who had not (P=.041). A total of 40.6% of participants who knew about epilepsy had good knowledge compared with the 13.3% of participants who had poor knowledge (P=.001) Additionally, participants who attended a course on seizure control (46.7%) had good knowledge in comparison to (31.2%) of those who did not (P=.037) (Table 4).

**Table 4:** Factors associated with participants’ knowledge regarding epilepsy among the study population P: Pearson X2 test; * P < 0.05 (significant); $: Exact probability test

Factors	Knowledge level				p-value
	Poor		Good		
	No	%	No	%	
Age in years					.388
18-30	132	66.0%	68	34.0%	
31-49	107	66.0%	55	34.0%	
50+	30	76.9%	9	23.1%	
Gender					.969
Male	81	66.9%	40	33.1%	
Female	188	67.1%	92	32.9%	
Marital status					.350
Single	124	63.6%	71	36.4%	
Married	133	70.4%	56	29.6%	
Divorced / widow	12	70.6%	5	29.4%	
Educational level					.848^$^
Below secondary	15	68.2%	7	31.8%	
Secondary	53	69.7%	23	30.3%	
University / above	201	66.3%	102	33.7%	
Employment					.004*
Not working / retired	73	72.3%	28	27.7%	
Student	68	55.3%	55	44.7%	
Working	128	72.3%	49	27.7%	
Monthly income					.644
< 5000 SR	56	71.8%	22	28.2%	
5000-10000 SR	79	69.9%	34	30.1%	
10001-15000 SR	56	62.9%	33	37.1%	
15001-20000 SR	40	62.5%	24	37.5%	
> 20000 SR	38	66.7%	19	33.3%	
Family history of epilepsy					.268
Yes	43	61.4%	27	38.6%	
No	226	68.3%	105	31.7%	
Heard about epilepsy					.048*
Yes	241	65.7%	126	34.3%	
No	28	82.4%	6	17.6%	
Know about epilepsy					.001*
Yes	171	59.4%	117	40.6%	
No	98	86.7%	15	13.3%	
Have you ever attended a course on seizure control?					.037*
Yes	24	53.3%	21	46.7%	
No	245	68.8%	111	31.2%	
Have you ever seen a patient suffer from an epileptic fit?					.089
Yes	143	63.6%	82	36.4%	
No	126	71.6%	50	28.4%	

## Discussion

The results provide common beliefs, practices, and awareness of first-aid management of seizures among the population in Makkah. Our aim was to utilize the results as a tool to measure the level of seizure awareness and assess any false beliefs or dangerous practices that may increase the risk of injury in patients with epilepsy. We report a total score of 32.9% of participants having a good experience with epilepsy and its treatment. The estimated score clearly indicates the insufficient ability of the public to deal with a patient having a seizure in the community. Nevertheless, the low awareness score in the current study was not unexpected. For example, only 32% of female teachers in primary schools in Riyadh expressed the ability to provide first-aid to their students with epilepsy. Additionally, 64% of male teachers in primary and intermediate schools in Southern Saudi Arabia were not able to provide first-aid to their epilepsy students during a seizure attack although they are usually exposed to some mandatory governmental training on first aid [13].

Epilepsy is a chronic neurological illness associated with several psychiatric and physical comorbidities. The negative effects of stigma, social detachment, and embarrassment because of seizures, and the failure to get proper education, get married, and get a job are additional impairments to the quality of life for many patients with epilepsy. A total of 13.5% of participants reported that epilepsy is a type of madness. In addition, 21.7% would not let their children associate with a person with epilepsy and would object to marrying a person with epilepsy. We observed a positive attitude regarding the right of epileptic patients to have an equal job opportunity from 77.1% of the participants. Also, the majority of participants thought women with epilepsy can have a normal pregnancy (76.1%), and that children with epilepsy can perform well in public school.

The current study displayed that there is a good level of knowledge regarding the etiology of epilepsy, as about 68.3% of participants answered neurological diseases when asked about the cause. These results are close to what was reported in studies from Alkharj [8], Riyadh [14], And Jeddah [7]. On the other hand, about 21.4% of individuals chose envy, and 12.7% chose evil spirits as a cause for epilepsy, which implies a lack of awareness about the disease's nature and etiology. This reflects a better public awareness of the causes of epilepsy compared to a study conducted by Al-Dossari et al. among the general population of the Alkharj region in 2021 [8]. In the Alkharj region, 46.5% of the respondents believed that epilepsy is caused by demonic possession or evil spirits, and more than half of respondents cited envy or evil eye. This is also consistent with previous studies coming from different regions of Saudi Arabia [15,16]. Around 73.6% of our participants believed that medication can help in the treatment of epilepsy, while 37.7% believed in faith and spiritual treatment with Ruqia and Quraan. Interestingly, 19.7% are not aware that treatment exists for epilepsy. It was noted that treatment with Ruqia and Quraan is widely used among Saudi Arabians [2[, and Arabian countries such as Kuwait [17] and United Arab Emirates [18]. A reasonable explanation for this is religious beliefs. When compared to other research conducted in different regions of Saudi Arabia [7,13,14], our findings revealed that Makkah city’s population is not much different in terms of the awareness of epilepsy treatments. Regarding the correct action during an epileptic fit, 82.1% of responders chose to call an ambulance and 55.1% knew that they should tilt the patient to the side position. Our results regarding the correct action during an epileptic fit reflect a better awareness in comparison to the results of a study done in Makkah with a total of 426 teachers to evaluate their knowledge of seizure first aid. In this case, the majority of teachers (55%) chose to open the patient's mouth and insert an object [19]. The overall participants’ knowledge regarding epilepsy and its treatment is poor among study participants in Makkah city, Saudi Arabia.

Respondents of our survey with a post-secondary school education had a higher average score of epilepsy knowledge than those with lower levels of education. An epilepsy education program or campaign could enhance participants' knowledge of and attitude toward epilepsy. The students of primary and secondary schools should be encouraged to get proper education and develop positive, supportive attitudes toward persons with epilepsy. This will be important and beneficial for future epilepsy care in Saudi Arabia [20].

This current survey represents an important extension of the previous studies done in Makkah city which were conducted only on physicians, teachers, and students. In our study we bridged the gap, by including the general population across different specialties, thereby strengthening the study, and allowing more representative results. 

Limitations

The purpose of this study was to assess the level of knowledge, attitude, and practices toward seizure attacks among the general population of Makkah city. Every effort to get reliable, satisfactory, and accurate results was made. However, some limitations were documented throughout this study's process. First, the study design was an online questionnaire; more accurate results could have been obtained with a physical form. Second, most of our study participants were female (69.8%). In addition, most of our participants were university undergraduates (75.6%). Therefore, further research is needed on this topic concerning different sociodemographic data and other study designs.

## Conclusions

This study revealed that most Makkah city residents had poor overall knowledge regarding epilepsy and seizure attacks. Attending an epilepsy education course is a predictor of improved overall knowledge and awareness of epilepsy. A health education program and awareness campaigns could help improve this knowledge gap in Makkah city.
